# Assessing the visual vertical: how many trials are required?

**DOI:** 10.1186/s12883-015-0462-6

**Published:** 2015-10-22

**Authors:** C. Piscicelli, S. Nadeau, J. Barra, D. Pérennou

**Affiliations:** Département de Rééducation Neurologique, Centre Hospitalier Universitaire de Grenoble, Grenoble, France; Laboratoire de Psychologie et Neurocognition CNRS UMR 5105, Grenoble Université, Grenoble, France; École de réadaptation, Université de Montréal and Centre de recherche interdisciplinaire en réadaptation (CRIR), Québec, Canada; Laboratoire Vision, Action, Cognition, Université Paris Descartes, Paris, EA7326 France

**Keywords:** Verticality perception, Spatial cognition, Stroke, Postural disorders, Repeatability, Measurement

## Abstract

**Background:**

The visual vertical (VV) consists of repeated adjustments of a luminous rod to the earth vertical. How many trials are required to reach consistency in this measure? This question has never been addressed despite the widespread clinical use of the measurement in stroke rehabilitation.

**Methods:**

VV perception was assessed (10 trials) in 117 patients undergoing rehabilitation after a first hemisphere stroke. The intraclass correlation coefficient (ICC) and standard error of measurement (SEM) were calculated for each patient category: with contralesional VV bias (*n* = 48), ipsilesional VV bias (*n* = 17) and normal VV (*n* = 52).

**Results:**

For patients with VV biases, 6 trials were required to reach high inter-trial reliability (contralesional: ICC = 0.9, SEM = 1.36°; ipsilesional: ICC = 0.896, SEM = 0.96°). For patients with normal VV, a minimum of 10 trials was required (ICC = 0.728, SEM = 1.13°). A set of 6 trials correctly classified 96 % of patients.

**Conclusions:**

In the literature, 10 is the most frequently used number of trials used to assess VV orientation. Our study shows that 10 trials are required to adequately measure VV orientation in non-selected subacute stroke patients. For complex protocols imposing a decrease in the number of trials in each condition, 6 trials are needed to identify VV biases in most patients.

## Background

Visual vertical (VV) measurement is the most commonly used test to identify altered verticality perception as a possible cause of postural disorders after stroke [1, 2]. The measurement consists of repeated adjustments of a luminous rod to the earth vertical in darkness from which the mean orientation perceived as vertical (VV orientation) is calculated [1–7].

After hemisphere stroke, biases of VV are mainly contralesional and are considered the consequence of a tilted verticality representation [8]. The main clinical manifestation of this contralesional bias of verticality perception is lateropulsion, an active lateral tilt of the body toward the side opposite the lesion [8]. Ipsilesional VV biases are less frequent (about 10 % of patients after hemisphere stroke) and smaller in magnitude [2, 3], and their cause remains to be clarified. Patients with a hemisphere stroke who show an ipsilesional VV bias never show an ipsilesional bias of the postural vertical [2]. The observation of an ipsilesional VV bias might be influenced by the measurement setting – the head and trunk maintained upright or free [9].

In estimating these alterations in VV perception, the number of adjustments reported in the published studies varies from 2 [5] to 30 trials [10]. The number of adjustments, usually even [1–7, 11, 12], counterbalances the leftward and rightward initial tilt to reduce the effect of the initial tilt position of the rod on VV measurement [13]. There is no consensus on the number of adjustments used to assess VV in clinical and research trials, although 10 is often used [2–4, 6, 7]. Moreover, studies of stroke patients assessing VV with more than 10 trials are few [10, 12] because these patients often present high fatigability and limited attentional resources, which may compromise or limit clinical assessments. This situation is particularly true for subacute stroke patients in neuro-rehabilitation units, where a reliable measure of VV perception is of primary interest for a better understanding of mechanisms underlying postural disorders and for follow-up of patients with a tilted VV perception. The interpretation of repetitive assessments for this clinical follow-up requires reliable measures.

The number of adjustments required to achieve inter-trial reliability for VV perception measurement is uncertain. We addressed this issue in the present study of subacute stroke patients with normal VV perception or contralesional or ipsilesional VV biases.

## Methods

### Patients

We performed a prospective observational study including 117 consecutive subacute stroke patients (62.1 ± 14 years; 48 females, 69 males; 60 left - and 57 right-sided lesions) admitted to a neuro-rehabilitation unit over 3 years and assessed for the first time in VV perception. All patients included had experienced a single recent (7.2 ± 6.6 weeks) haemorrhagic (20) or ischemic (97) hemisphere stroke without any selection regarding the affected arterial territory. We excluded patients with neuropathy, psychiatric disorders, or major comprehension problems due to aphasia or dementia and those with unstable medical problems. The study was performed in compliance with the Helsinki Declaration. All subjects provided informed consent. According to French law, as a non-interventional (observational) study, this research did not require approval by an ethics committee.

### VV assessment

The assessment procedure was as previously described by Pérennou et al. [2]. VV perception was assessed in darkness by binocular visual adjustment of the direction of a bright line (15 cm long, 2 mm wide) presented on a computer screen at eye level. For patients with hemineglect or hemianopia, the computer screen could be moved a few centimeters so that the patient saw the entire line. After 2 training trials, 10 adjustments were performed with each patient, in agreement with the literature [2–4, 6, 7] and to reduce patients’ fatigability. The initial orientation of the line, ranging from 5 to 30°, was randomly determined and the tilts of the line were presented in a fixed sequence balanced between leftward and rightward. To avoid any bias due to the setting [2, 7, 9, 14], patients were seated with their head and trunk maintained upright and straight by using lateral cushioning blocks. They were asked to verbally adjust the line to the vertical. There was no time limit or feedback from the examiner. The measures started after 2 min of darkness.

For each patient, we calculated the mean VV orientation [1–4, 6, 7] over the 10 trials. Consistent with standards, after sign transformation according to the lesion side, a negative value corresponded to a contralesional bias. Patients were classified as having normal VV perception (from −2.5 to 2.5°) or contralesional (<−2.5°) or ipsilesional (>2.5°) bias according to normal ranges validated in a previous study [2].

### Statistical analysis

Descriptive statistics with mean ± SD when relevant were used to characterize the study population. One-way ANOVA and chi-square statistics were used to compare the characteristics of the three patient subgroups: normal VV perception or contralesional or ipsilesional bias. VV orientation was statistically analyzed for each subgroup (contralesional VV bias, ipsilesional VV bias, normal VV perception). The distribution of VV orientation for the 10 trials were normal in each subgroup by Kolmogorov-Smirnov test. Inter-trial reliability, the consistency of the measures, was assessed for the first 2, 4, 6, 8, and 10 trials in each subgroup, in agreement with the literature [13]. Relative reliability was quantified by the intraclass correlation coefficient (ICC) [15]. The standard error of measurement (SEM = SD × √1 − ICC), indicating the precision of the measure, was expressed in degrees. The lowest number of trials meeting the criterion ICC ≥0.9 indicated the minimum number of trials required for high inter-trial reliability of VV perception. For each even number of trials (2, 4, 6 and 8), the number (percentage) of patients classified into subgroups (normal, contralesional or ipsilesional bias) other than the one established for 10 trials was calculated. Statistical analyses involved use of SPSS 21 (IBM Corp., Armonk, NY, USA).

## Results

**Fig. 1:**
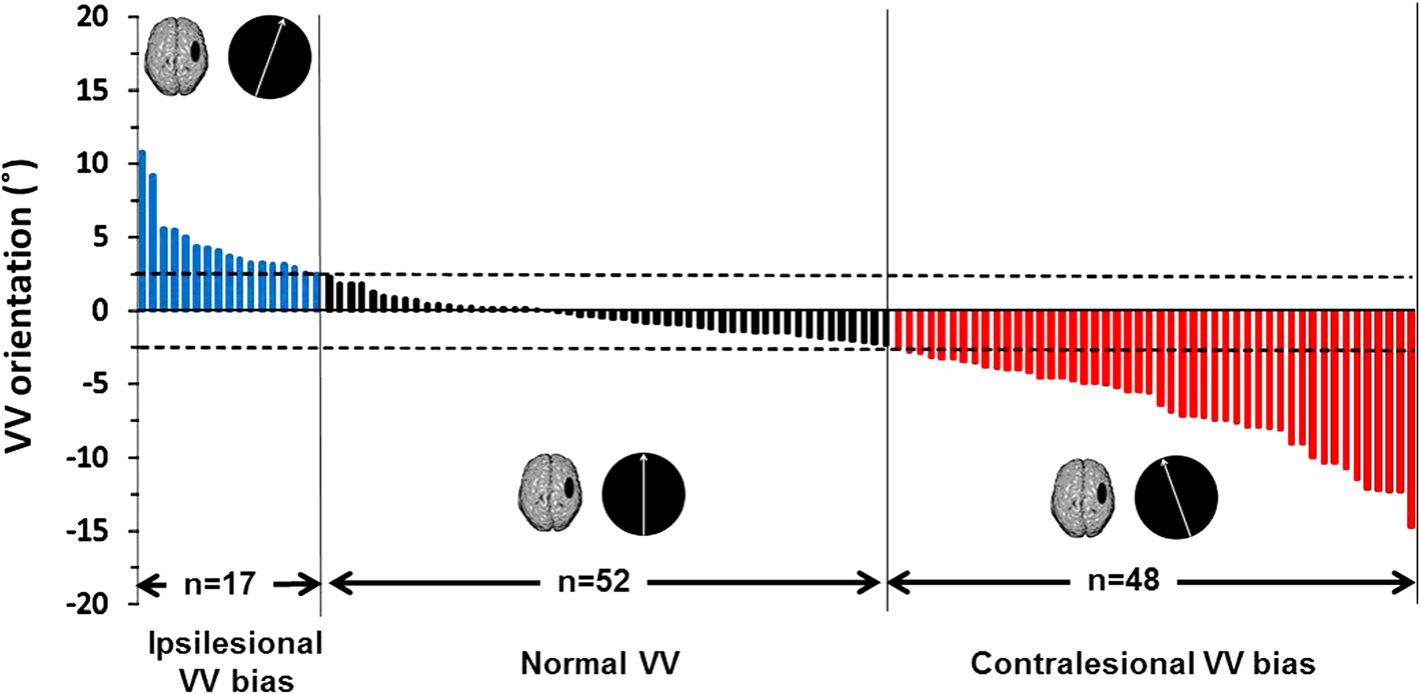
Visual vertical (VV) perception in patients with subacute stroke. VV orientation estimated in 10 trials for 117 subacute stroke patients with ipsilesional VV bias, normal VV perception and contralesional VV bias according to normal ranges (−2.5°; 2.5°) [2] indicated by dashed lines

Among the 117 stroke patients, VV perception was normal for 52 (44.4 %) (−0.46° ± 1.1) and was contralesionally tilted for 48 (41 %) (−6.7 ± 3.1°) and ipsilesionally tilted for 17 (14.5 %) (4.5 ± 2.3°) (Fig. 1). This pattern corresponds to that reported in the literature [2, 3]. The three patient subgroups did not differ in age (F(2, 114) = 1.026, *p* = 0.36), time since stroke (F(2, 114) = 0.36, *p* = 0.69), gender (*p* >0.05) or stroke etiology (*p* >0.05). They differed in lesion side, with more right-sided lesions in the contralesional bias than the normal VV subgroup (*χ*2 = 8.79, *p* = 0.003); the others did not differ (all *ps* >0.09).

### Inter-trial reliability for patient subgroups

**Fig. 2:**
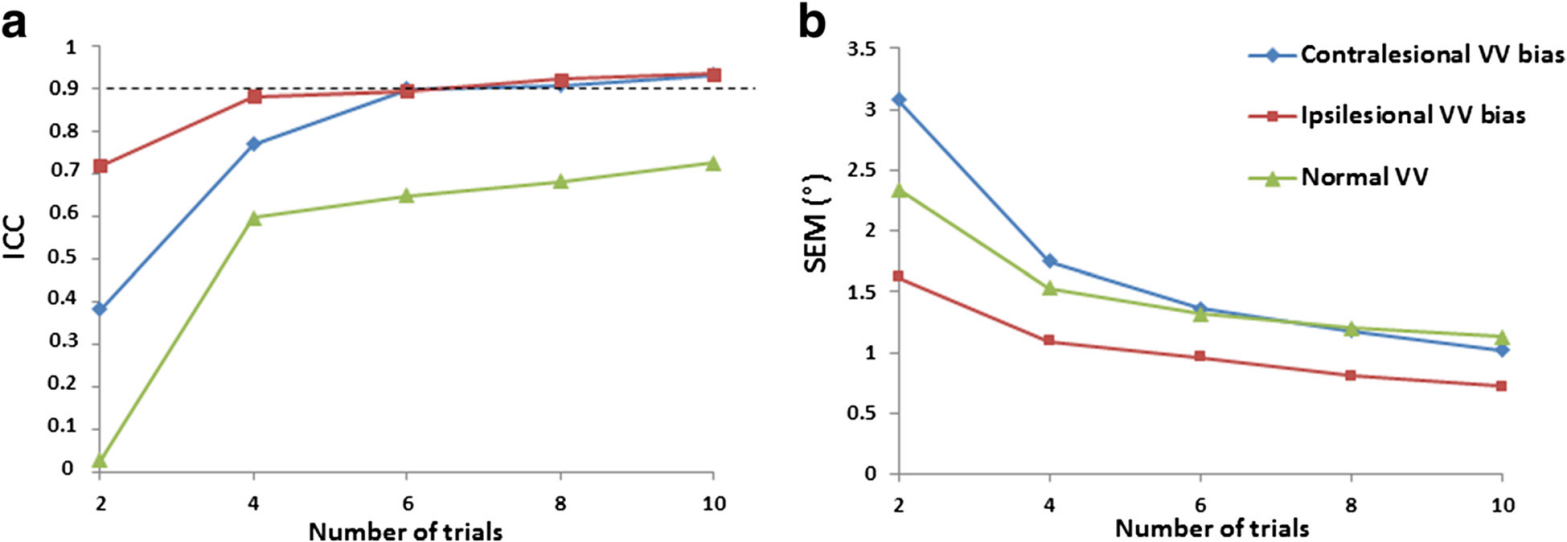
Inter-trial reliability by number of trials and patient subgroups. **a** Intraclass correlation coefficients (ICCs) and (**b**) standard error of measurement (SEM, in degrees) for 2, 4, 6, 8 and 10 trials for patients with contralesional VV bias (*n* = 48), ipsilesional VV bias (*n* = 17) and normal VV perception (*n* = 52). In (**a**), the dotted line represents ICC ≥0.9 indicating high inter-trial reliability

For the 48 patients with contralesional VV bias, the ICC and SEM ranged from 0.382 to 0.933 and 3.08 to 1.02°, respectively (Fig. 2). For contralesional VV bias, assessment required 6 trials to obtain reliable VV orientation and the SEM was 1.36°. Corresponding values for the 17 patients with ipsilesional VV bias were 0.721–0.935 and 1.62–0.72°, respectively (Fig. 2). For ipsilesional VV bias, assessment required a minimum of 6 trials to obtain reliable VV orientation, with a SEM of 0.96°. For the 52 patients with normal VV perception, the ICC and the SEM ranged from 0.029 to 0.728 and 2.34 to 1.13°, respectively (Fig. 2). For normal VV perception, assessment required >10 trials to obtain an ICC ≥0.9. The SEM was 1.13° with 10 trials.

### Concordance of patient classification based on number of trials

When VV orientation was estimated with 2 and 4 trials, 88 % (103/117) and 92 % (108/117) of patients were classified in the same subgroup as with 10 trials. With 6 and 8 trials, four patients were misclassified, for 96 % concordance of classification.

## Discussion

The aim of this study was to determine how many trials are required to assess VV perception after stroke to better adapt assessment to patient abilities and at the same time maintain good inter-trial reliability of the measure. Surprisingly, our study is the first to address the question of the number of trials sufficient for VV measurement.

To the best of our knowledge, among the myriad studies of VV perception using different numbers of trials, no studies have ever justified the choice of this number. Our study showed that the number of trials needed to reach high inter-trial reliability was a minimum of six for patients with contralesional and ipsilesional biases. For this number of trials, the SEM was slightly higher than 1° for the contralesional bias subgroup. For patients with normal VV perception, it seems that more than 10 trials are required, but the SEM (1.13°) with this number was as good as for the other subgroups. As compared with 10 trials, 6 and 8 trials allowed for a correct identification of alterations in VV perception in most patients, with less than 5 % of the patients misclassified.

These findings are valid for the conditions under which VV was assessed in our study, (i.e., VV assessed with the method of adjustments [13] in subacute hemispheric stroke patients seated, with the trunk and head maintained upright [9]) and for a fixed criterion (ICC ≥0.9), guaranteeing, in our opinion, a high reliability of the measure in clinical practice.

In our study, the minimum number of trials identified was the same for the two subgroups of patients with biased verticality perception (contralesional or ipsilesional), even if the underlying mechanisms of these biases are probably different [2]. However, for similar SEMs for these two VV alterations, more trials (up to 10) are needed for patients presenting contralesional bias.

In patients with normal VV perception, VV orientation assessment required more than 10 trials to reach the ICC ≥0.9 cutoff. The lowest ICCs for this subgroup are certainly affected by the lower inter-subject variance (to −2.37° from 2.31°) as compared with other subgroups (to −14.72° from −2.61° and to 2.51° from 10.79° for contralesional and ipsilesional subgroups, respectively). Because ICC values are a ratio of the variance between subjects and the total variance, coefficients are systematically lower in homogeneous samples [16]. This argument is supported by the correct SEM (1.13°) value with 10 trials for this subgroup. Considering the large size of this subgroup (*n* = 52) and that the inter-subject variance is limited by two boundaries, more than 10 trials may not contribute to significantly increasing the ICC.

### Study limitations

In our study, participants adjusted the luminous rod without a time limit as is usual in clinical practice. This situation respects both the capacity and strategy of every patient. However, if healthy participants spend more time on single adjustments, the trial becomes more accurate and precise [17]. Since the aim of this study was to generalize findings for clinical use in stroke rehabilitation, the procedure was similar to what we use in clinical practice.

We selected 10 trials as the maximum number of trials to assess VV perception in agreement with the literature on VV perception in stroke patients [2–4, 6, 7, 9] and to limit examination time due to fatigability in stroke patients. Fatigability was not quantified because the use of questionnaires is difficult with hemisphere stroke patients who frequently present aphasia and other cognitive disorders. However, the examiner ensured that patients felt able to complete the assessment and that their response did not become aberrant.

## Conclusion

According to the context of assessment and patients’ profiles, 6–10 trials are needed to obtain reliable VV orientation in subacute stroke patients.

## Abbreviations

VV: Visual vertical
ICC: Intraclass correlation coefficient
SEM: Standard error of measurement
